# In Vivo Fluorescence Imaging of Passive Inflammation Site Accumulation of Liposomes via Intravenous Administration Focused on Their Surface Charge and PEG Modification

**DOI:** 10.3390/pharmaceutics13010104

**Published:** 2021-01-14

**Authors:** Hisako Ibaraki, Akihiro Takeda, Naoki Arima, Naruhiro Hatakeyama, Yuuki Takashima, Yasuo Seta, Takanori Kanazawa

**Affiliations:** 1 School of Pharmacy, Tokyo University of Pharmacy and Life Sciences, 1432-1 Horinouchi, Hachioji, Tokyo 192-0392, Japan; ibaraki@toyaku.ac.jp (H.I.); ii.iv.vi.2.4.6.not.1.3.5@gmail.com (A.T.); n.arima@ono.co.jp (N.A.); naru.hatakeyama@gmail.com (N.H.); takasima@toyaku.ac.jp (Y.T.); setayas@toyaku.ac.jp (Y.S.); 2 School of Pharmaceutical Sciences, University of Shizuoka 52-1 Yada, Suruga-ku, Shizuoka 422-8526, Japan

**Keywords:** nanomedicine, drug delivery system, liposome, polyethylene glycol, rheumatoid arthritis, ulcerative colitis, in vivo imaging

## Abstract

Nanocarriers such as liposomes have been attracting attention as novel therapeutic methods for inflammatory autoimmune diseases such as rheumatoid arthritis and ulcerative colitis. The physicochemical properties of intravenously administered nanomedicines enable them to target inflamed tissues passively. However, few studies have attempted to determine the influences of nanoparticle surface characteristics on inflammation site accumulation. Here, we aimed to study the effects of polyethylene glycol (PEG) modification and surface charge on liposome ability to accumulate in inflammatory sites and be uptake by macrophages. Four different liposome samples with different PEG modification and surface charge were prepared. Liposome accumulation in the inflammation sites of arthritis and ulcerative colitis model mice was evaluated by using in vivo imaging. There was greater PEG-modified than unmodified liposome accumulation at all inflammation sites. There was greater anionic than cationic liposome accumulation at all inflammation sites. The order in which inflammation site accumulation was confirmed was PEG-anionic > PEG-cationic > anionic > cationic. PEG-anionic liposomes had ~2.5× higher fluorescence intensity than PEG-cationic liposomes, and the PEG-liposomes had ~2× higher fluorescence intensity than non-PEG liposomes. All liposomes have not accumulated at the inflammation sites in healthy mice. Furthermore, cationic liposomes were taken up to ~10× greater extent by RAW264.7 murine macrophages. Thus, PEG-cationic liposomes that have the ability to accumulate in inflammatory sites via intravenous administration and to be taken up by macrophages could be useful.

## 1. Introduction

Inflammatory diseases include allergic diseases such as asthma and atopic dermatitis and autoimmune diseases such as rheumatoid arthritis (RA), inflammatory bowel disease (IBD), and myasthenia gravis. Numerous disorders and conditions are characterized by inflammation. Autoimmune diseases include more than 80 different chronic conditions that affect nearly 5% of the human population, and for reasons that are poorly understood, the incidence and prevalence of autoimmune diseases are rising [1]. They are characterized by immune responses that damage or cause dysfunction to specific or multiple organs and tissues. The etiology of autoimmune disease is still unknown. However, genetic, environmental, and lifestyle factors may contribute to its development [2,3]. Treatment for autoimmune diseases such as RA and ulcerative colitis is limited to symptomatic management with corticosteroids and immunosuppressants, which also cause a substantial deterioration in the quality of life [4,5,6].

In recent years, the satisfaction of treatment for these diseases has been improving with antibody drugs. Anti-human TNF-α monoclonal antibody infliximab (Remicade^®^, Janssen Pharmaceutical K.K.), Adalimumab (HUMIRA^®^ AbbVie Inc., Chicago, IA, USA), Vedolizumab (Entyvio^®^, Takeda Pharmaceutical Company Limited, Osaka, Japan) for other patients with severe autoimmune disease have become epoch-making treatments [7,8,9,10]. However, antibody drugs tend to require enormous costs (for example, Entyvio^®^: $4000), time, and labor due to biological preparations. Additionally, serious side effects are still a big problem [11,12]. Therefore, the development of new therapeutic methods using nanomedicines has attracted attention and is urgently needed. In addition, nanomedicine and nanotechnology are expected to be applied not only in treatment but also in a wide range of applications such as diagnosis and prognosis [13,14].

The physicochemical properties of nanomedicines are closely associated with their pharmacokinetics and tissue accumulation levels [15]. In the enhanced permeation and retention (EPR) effect, molecules with a molecular weight higher than 40 kDa leak towards tumor tissue margins because of angiogenesis and enhanced vascular permeability in cancer tissue. Hence, the substances passively accumulate in the tumors [16]. The EPR effect plays a central role in nanomedicine-based anticancer drug delivery systems. The EPR effect may also occur in inflammatory sites as vascular permeability is observed in the acute phase, and the formation of new blood vessels is observed in the chronic phase of inflammation. Recently, the EPR effect was detected in inflammation associated with pathological tissues characteristic of pulmonary arterial hypertension and in an inflammatory bowel disease model [17,18].

Intravenous administration of nanocarriers such as liposomes, nano-micelles, and dendrimers are efficacious in inflammatory disease treatments [19,20,21]. Liposomes are widely used as they have low immunogenicity, and their surface properties can be easily modulated. The physicochemical properties of liposomes enable them to exploit the passive targeting function provided by the leaky vasculature of the inflamed tissue. The passive targeting capacity of liposomes or nanocarriers may depend primarily on enhanced blood circulation time, which, in turn, is affected by vesicle size and surface charge and hydrophobicity [22,23,24,25].

We previously reported that intravenous administration of the block-polymer micelle MPEG-PCL-CH2R4H2C and the cytoplasm-sensitive peptide STR-CH2R4H2C had strong therapeutic efficacy against inflammatory diseases such as RA and atopic dermatitis, and cancer [26,27,28,29,30,31,32,33,34,35,36,37]. These molecules were designed in our laboratory as novel drug carriers. We also indicated that liposomal surface properties are strongly affected to improve the delivery efficacy for the treatment of atopic dermatitis and the activity against biofilm [34,35,36]. However, few studies have comprehensively investigated the physical properties of intravenously administered nanocarriers that are highly effective at treating inflammatory diseases. Hence, our aim here was to determine the relative efficacy and optimal combination of surface charge and polyethylene glycol (PEG) modification of liposomes for the treatment of RA and ulcerative colitis via intravenous administration, and evaluated the characteristics enabling liposomes to accumulate in the inflammation site. We compared four liposome samples with different surface charges and PEG modification by intravenously administering them to induced RA or ulcerative colitis mouse models. Finally, the uptake of each liposome by RAW264.7 murine macrophages associated with inflammatory responses was measured.

## 2. Materials and Methods

### 2.1. Materials, Cells, and Mice

The 1,2-dioleoyl-sn-glycero-3-phosphoethanolamine (DOPE), 1,2-dioleoyl-3-trimethylammonium-propane (DOTAP), and 1,2-distearoyl-sn-glycero-3-phosphoethanolamine-N-[methoxy (polyethylene glycol)-2000] (DEPE-PEG2000) were purchased in dry powder format from Avanti Polar Lipids (Alabaster, AL, USA). Cholesteryl hemisuccinate (CHEMS) was purchased from Sigma-Aldrich Corp. (St. Louis, MO, USA). The 1,2-dioleoyl-sn-glycero-3-phosphoethanolamine ATTO 647N (ATTO-DOPE) used in fluorescein labeling of liposome lipid films were obtained from ATTO-TEC (Siegen, Germany). The rotary evaporator system used to prepare the liposomes consisted of a CCA-1111 recirculating chiller, a DPE-1120 solvent recovery unit, an NVC-2100 vacuum controller, an N-1100 rotary evaporator, an OSB-2100 water bath (EYELA, Tokyo, Japan), a DIVAC 1.2 L vacuum pump (Oerlikon, Pfäffikon, Switzerland), and a probe-type sonicator.

Eight-week-old male DBA/1J mice and seven-week-old female C57BL/6 mice were purchased from SLC (Shizuoka, Japan). All animal experiments were conducted out in accordance with protocols (Nos. P14-02 and P15-65) approved by the Animal Care and Ethics Committee of Tokyo University of Pharmacy and Life Sciences, Tokyo, Japan. The mice were housed at 23.5 ± 1 °C, 55 ± 5% relative humidity (RH), and a 12 h light/dark cycle. Bovine type II collagen (CII; Life Laboratory Co., Yamagata, Japan), complete Freund’s adjuvant (CFA; MP Biomedicals LLC, Irvine, CA, USA), or incomplete Freund’s adjuvant (IFA; MP Biomedicals LLC, Irvine, CA, USA) was used to prepare a RA model mouse. Reagent grade dextran sulfate sodium salt (DSS) (MW 36,000–50,000 (IFA; MP Biomedicals LLC, Irvine, CA, USA) was used to prepare an ulcerative colitis model mouse. RAW264.7 murine macrophages were obtained from the American Type Culture Collection (ATCC; Manassas, VA, USA). Dulbecco’s modified Eagle’s medium (DMEM; Nacalai Tesque, Kyoto, Japan), fetal bovine serum (FBS; Life Technologies, Carlsbad, CA, USA), and penicillin (10,000 U mL^−1^)–streptomycin (10,000 g mL^−1^) solution (Nacalai Tesque, Kyoto, Japan) were used in the cytological assays.

### 2.2. Liposome Preparation and Physical Property Measurement

Liposomes were prepared using the thin film method [38] shown in Table 1. A total of 3 μmol of the fluorescence-labeled lipids ATTO-DOPE and DOPE was used. The mol% of DOPE, which was the primary lipid in all liposomes, was 46.15 for PEG-cationic liposomes, 54.54 for PEG-anionic liposomes, 50.00 cationic liposomes, and 60.00 for anionic liposomes. The lipid powders were dissolved in 5 mg mL^−1^ chloroform, and the solutions were stored at −20 °C before use according to Avanti polar lipids’ recommended storage temperature. The lipid solutions were placed in a glass tube according to Table 1 and subjected to rotary evaporation to prepare thin lipid films. Then 10 mM HEPES buffer (pH 7.4) was added to hydrate the lipid films, and the mixture was vortexed. The mixture was sonicated and then subjected to 20 s probe-type ultrasonication. It is a well-known report that probe-type sonication turns a multilamellar vehicle (MLV) into a small unilamellar vehicle (SUV) and reduces the liposome size [39]. The popular method for the preparation of SUV.

The sizes and the zeta potentials of the empty liposomes were measured using a Zetasizer Nano (Malvern P Analytical, Malvern, UK). The size was determined by the dynamic light scattering method, and the zeta potential was measured by the electrophoretic light scattering method.

### 2.3. Preparation of Collagen-Induced Arthritis Mice

The collagen-induced arthritis (CIA) mouse model is the most widely used model to study RA. Arthritis in this model is triggered by autoantibodies against type II collagen [40]. CIA mouse model was developed as previously reported [29]. Adjuvant (CFA or IFA) and CII solutions were added dropwise to the adjuvant under ice-cooling to prepare an emulsion; then, the CII + CFA emulsion was intradermally administered to 8-week-old male DBA/1 J mice at the ridge for initial immunization. After 21 days, the CII + IFA emulsion was intradermally administered for booster immunization. Arthritis severity was evaluated by using the clinical scoring method [29]. Briefly, the front paw was assigned 0.5 points per swelling per finger and one point for erythema and swelling of the entire paw. Hence, the maximum possible clinical score for each front paw was three. The rear paw was assigned 0.5 points per swelling per finger, one point for erythema and swelling of the entire paw, and one point for erythema and swelling of the ankle joint. Thus, the maximum possible clinical score for each rear paw was 4.5. Mice with clinical scores > 3.5 were defined as having RA.

### 2.4. Evaluation of Inflammatory Site Accumulation Using In Vivo Imaging after Intravenous Liposome Administration in CIA Mice

Two hundred microliters of each liposome (ATTO-DOPE: 0.01 µmol/200 µL or 0.1 µmol/200 µL) was intravenously administered to CIA mice. After 0.5–12 h, ATTO-DOPE accumulation in the inflamed paws was evaluated by observing ATTO-DOPE fluorescence localization with an in vivo imaging system (Maestro^TM^; PerkinElmer Inc., Waltham, MA, USA). The fluorescence intensity was also evaluated with Maestro™ software.

### 2.5. Evaluation of Inflammatory Site Accumulation Using In Vivo Imaging after Intravenous Liposome Administration in an Ulcerative Colitis Mouse Model

An ulcerative colitis mouse model can be induced by oral dextran sodium sulfate (DSS) administration [41,42]. Seven-week-old female C57BL/6 mice freely ingested 3% (*w*/*v*) DSS for 6 d and freely consumed tap water on day 7. Then 100 µL of each liposome (ATTO-DOPE: 0.04 µmol/100 µL) was intravenously administered to the DSS mice. After 1 h, 3 h, and 6 h, the large intestine was excised and washed with physiological saline. Fluorescent ATTO-DOPE localization in the large intestine was observed with an in vivo imaging device (Maestro™; PerkinElmer Inc., Waltham, MA, USA). The fluorescence intensity was also evaluated with Maestro™ software.

### 2.6. Evaluation of the Uptake Efficiency of Each Liposome by RAW264.7 Macrophages

Murine macrophages, RAW264.7 cells (5 × 10^5^) in DMEM with FBS (10% *v*/*v*) were precultured in 24-well plates at 37 °C under a humidified 5% CO_2_ atmosphere. After 24 h incubation, the cells were washed with phosphate-buffered saline (PBS) and transfected with PEG-cationic and PEG-anionic liposomes. Six hours after transfection, the RAW264.7 macrophages were washed with PBS, and their liposome uptake levels were measured using flow cytometry (BD FACS Canto; BD Biosciences, Franklin Lakes, NJ, USA). Intracellular uptake efficiency and average fluorescence intensity per cell were calculated.

### 2.7. Statistical Analysis

Statistical analysis of the data was performed using analysis of variance followed by t-test. Statistical significance was defined as highly significant (* *p* < 0.05, ** *p* < 0.01).

## 3. Results

### 3.1. Physical Properties of Various Liposomes

In this study, the effect of liposome surface properties on the accumulation of inflammatory sites via intravenous administration was evaluated from the viewpoint of surface charge and PEG modification for the treatment of inflammatory diseases. The liposomes were prepared using the thin film method, and the formulations are listed in Table 1. The data represents the mean size of the liposome samples prepared (*n* = 3). Their particle sizes and zeta potentials were measured. Cationic lipid DOTAP conferred a positive surface charge, and the cholesterol derivative CHEMS created a negative surface charge. Table 2 shows that all particles were ≤90 nm, and they were reduced by PEG modification. All subsequent experiments were conducted on the four liposome samples.

### 3.2. In Vivo Imaging of Inflammatory Sites after Intravenous Liposome Administration to CIA Mice

**Figure 1:**
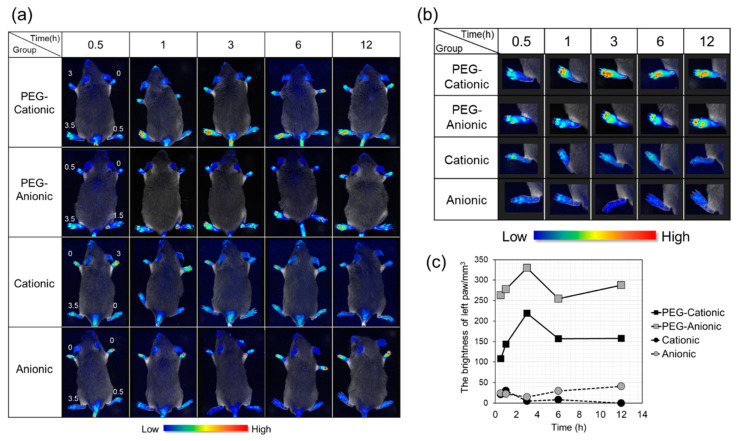
In vivo imaging and liposome brightness in collagen-induced arthritis (CIA) mice. CIA mice were intravenously administered four different liposome samples using Maestro^TM^. (**a**) Liposome accumulation in the inflammatory site. Numbers indicate clinical scores. (**b**) Extended image of the paw with clinical score = 3.5. (**c**) Brightness of left paw (clinical score = 3.5) determined by Maestro^TM^ v. 2.4.

Figure 1a,b shows in vivo liposomal localization, and Figure 1c show the fluorescence intensity in the paws. A high clinical score means that inflammation was promoted. Confirmation of fluorescence at a site with a high clinical score indicated liposome accumulating at the inflammation site. We evaluated the effects of liposome surface charge and PEG modification on inflammation site accumulation after intravenous nanoparticle administration in CIA mice. The numbers on the paws are the clinical scores. The body length of the model mouse was about 7–8 cm.

PEG-modified liposomes accumulated at the inflammation site to a greater extent than the unmodified liposomes (Figure 1a,b). Figure 1b shows a paw with a high clinical score, and Figure 1c illustrates that the fluorescence intensity of PEG-modified liposomes was four-fold higher than that of the unmodified liposomes.

Regarding the surface charge, cationic and anionic liposomes accumulated to similar levels at the inflammation sites (left paw; high clinical score) (Figure 1a,b). The semiquantitative analysis shown in Figure 1c discloses that PEG-anionic liposomes had ~2.5× higher fluorescence intensity than PEG-cationic liposomes. The subsequent experiments were performed based on the foregoing findings.

### 3.3. Effects of Liposome Surface Charge on Inflammatory Site Accumulation after Intravenous PEG-Modified Liposome Administration to CIA Mice

We confirmed the effectiveness of PEG modification at enhancing liposome accumulation at the inflammation site (Figure 1). However, there was concern that the clinical scores of the other paws were not the same. Therefore, in this section, we compared intravenously administered, PEG-modified liposome accumulation in normal mice and at the inflammation sites of CIA mice whose paw clinical scores were set to zero. Additionally, the fluorescently labeled lipid content was enhanced by approximately 10-times to improve visibility.

**Figure 2:**
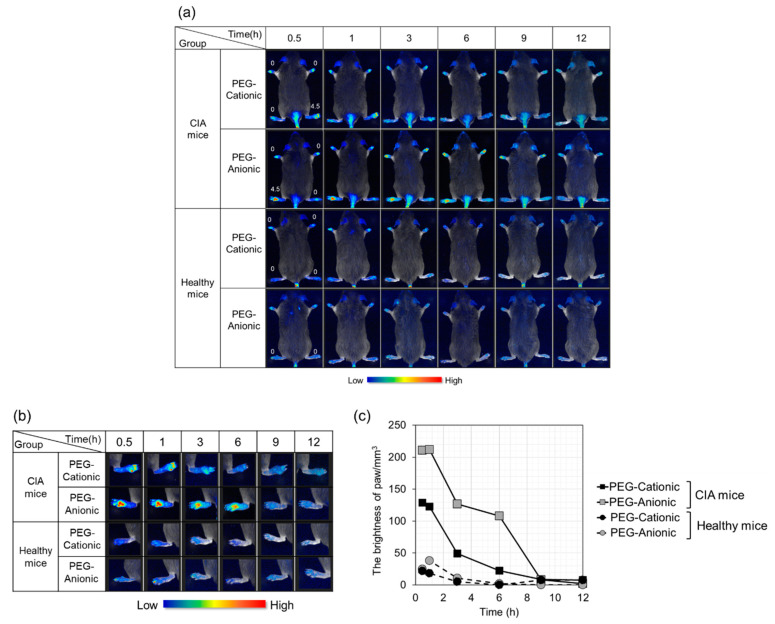
In vivo imaging and brightness of polyethylene glycol (PEG)-modified liposomes in CIA and healthy mice. (**a**) Liposome accumulation in the inflammatory site. Numbers indicate clinical scores. (**b**) Extended image of the paw with clinical score = 4.5. (**c**) Brightness of paws (clinical score = 4.5) determined by Maestro^TM^ v. 2.4.

In vivo, imaging is shown in Figure 2a,b. Figure 2b illustrates a foot presenting with an inflammatory reaction of Figure 2a. The fluorescence intensities of the hind paws with clinical score = 4.5 are shown in Figure 2c. In healthy mice, slight fluorescence indicated a moderate accumulation of every liposome. In contrast, clear fluorescence was confirmed in CIA mice. Therefore, inflammatory site-specific accumulation of liposomes was confirmed. The fluorescence in the paws with a score of 0 for PEG-liposomes was comparable to autofluorescence.

It was also indicated that anionic liposomes accumulated in the inflammation site to a greater extent than cationic liposomes (Figure 2a,b). The brightness analysis in Figure 2c reveals that the PEG-anionic liposome fluorescence intensity was about double that of PEG-cationic liposomes at the inflammation sites of the CIA mouse. The aforementioned findings indicate that liposomes most effectively accumulate at the inflammation site via intravenous administration when they are modified with PEG and have anionic surface charges.

### 3.4. In Vivo Imaging Evaluation of Inflammatory Sites in DSS Mice after Intravenous Liposome Administration

**Figure 3:**
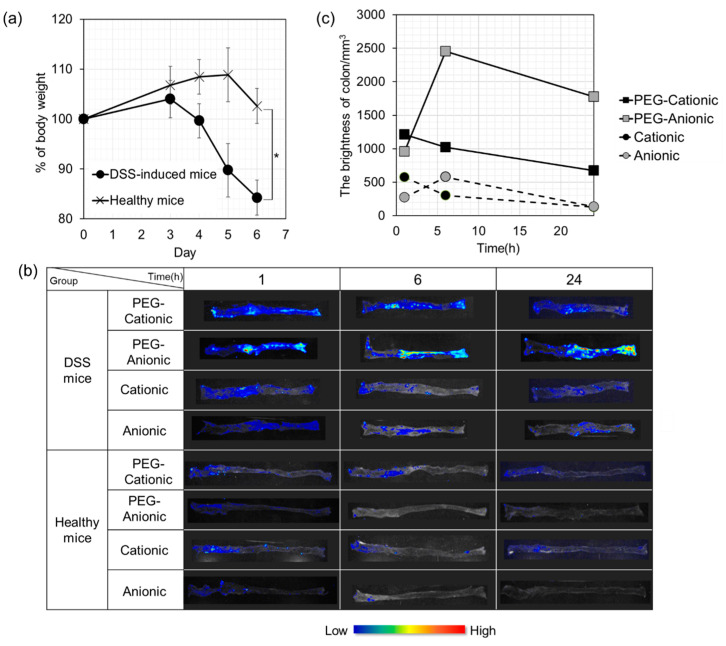
Accumulation of various liposomes in dextran sulfate sodium salt (DSS)-induced mice. (**a**) Weight loss in mice orally administered DSS. Each bar represents mean ± S.D (DSS-induced mice, *n* = 24; healthy mice, *n* = 15). (**b**) In vivo imaging of colon in response to intravenous liposome administration. (**c**) Brightness of various liposomes in DSS-induced mouse large intestines determined by Maestro^TM^ v. 2.4.

The dextran sulfate sodium (DSS)-induced ulcerative colitis model is widely used as it is simple and resembles human ulcerative colitis. We used in vivo imaging to evaluate the colonic accumulation of liposomes that had been intravenously administered to DSS mice (Figure 3). As shown in Figure 3a, loss in body weight and hematochezia were observed starting Day 4. Hence, we assessed liposome accumulation at the inflammation site 7 days after DSS administration. The length of the large intestine with ulcerative colitis was about 5–6 cm in Figure 3c.

All four liposomes were intravenously administered to both DSS-induced and healthy mice. The large intestines were excised and observed using an in vivo imaging system to confirm liposome accumulation in inflamed large intestine tissue. In healthy mice, no liposomes accumulated in the large intestine regardless of PEG modification or surface charge. For the DSS-induced mice, the unmodified liposomes did not produce any fluorescence in the large intestine. In contrast, PEG-modified cationic and anionic liposomes accumulated in the inflamed colon after 6 h. There was a peak in cationic liposome accumulation in the inflamed colon at 1 h after intravenous administration. However, there was a maximum anionic liposome accumulation at the colonic inflammation site at 3 h after intravenous administration. Strong fluorescence was detected there even after 24 h. One possible explanation is the high blood relative retention of anionic liposomes, and a similar finding was noted for the CIA mice (Section 3.3).

### 3.5. Evaluation of PEG Liposome Uptake by RAW264.7 Cells

**Figure 4:**
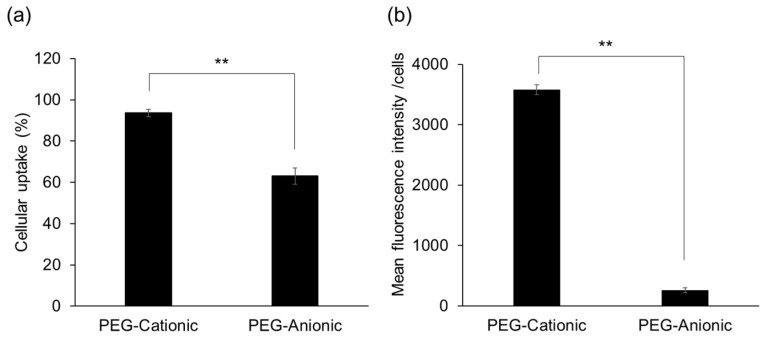
Cellular uptake of PEG liposomes in RAW264.7 murine macrophages. (**a**) Liposomal cellular uptake (%) in treatment compared to control. (**b**) Fluorescent intensity per cell per liposome in RAW264.7 macrophages. Each bar represents the mean ± S.D. (*n* = 3). ** *p* > 0.01. Fluorescent-labeled PEG-cationic and PEG-anionic liposomes transfected into RAW264.7 macrophages for 6 h. Samples were measured by flow cytometry.

Intracellular uptake efficiency of PEG-cationic and anionic liposomes by mouse macrophages was evaluated using flow cytometry. Figure 4 shows that PEG-cationic liposomes were far more effectively absorbed by RAW264.7 cells than PEG-anionic liposomes. The mean PEG-cationic liposome fluorescence intensity per cell was tenfold higher than that of PEG-anionic liposomes. Thus, it is clear that PEG-cationic liposome is effectively uptake into macrophages.

## 4. Discussion

Nanotechnology has attracted attention as a novel therapeutic approach towards the treatment of autoimmune inflammatory diseases such as RA and ulcerative colitis. Additionally, it is also expected to be used for diagnosis and prognosis, which enables early diagnosis of diseases and improvement of therapeutic effect. In general, disease-modifying anti-rheumatic drugs (DMARDs) and non-steroidal anti-inflammatory drugs (NSAIDs) have been administered for RA, and ulcerative colitis has been treated with 5-aminosalicylic acid (5-ASA), corticosteroids, and so on. Furthermore, not only antibody drugs that have become mainstream for severe patients, but also antisense oligonucleotides and nucleic acid drugs such as siRNA, miRNA, and aptamer that utilize RNA interference are groundbreaking agents for inflammatory diseases as a novel drug modality [32,43,44]. There are some reports that nanoparticles such as liposomes, polymeric micelles, dendrimers, exosomes, which were loaded these modalities have been intravenously administered, and these improve therapeutic efficacy and attenuate side effects by enhancing active or passive target tissue accumulation [18,27,28,29,30,37]. However, few studies have comprehensively analyzed what kind of physical property of nanomedicine in the treatment of inflammatory disease by intravenous administration contributes to the accumulation of inflammatory disease and the therapeutic effect. Therefore, in the present study, we prepared four types of liposomes, which was controlled PEG modification and surface charge, and their inflammation site accumulation by in vivo imaging system, and macrophage uptake properties by flow cytometry were evaluated.

We prepared PEG-cationic, PEG-anionic, cationic, and anionic liposomes using the thin-film method (Table 1). Their physical properties are listed in Table 2 (Section 3.1). Generally, nanoparticles with a diameter >200 nm have reduced blood retention and increased splenic accumulation after intravenous administration. Hence, they are easily recognized by the reticuloendothelial system (RES) [45,46]. Similarly, the surface charge of liposomes plays an important role in immune cell recognition and uptake by the RES [47]. Additionally, RA inflammation causes submicron-sized synovial endothelial vascular cell gaps [48] that are unimpeded by infiltrative gaps in leaky synovial vessels. Therefore, all four liposome types here were expected to accumulate at the inflammation sites after intravenous administration from the viewpoint of their size. Additionally, both cationic and anionic liposomes reduced the particle sizes via PEG modification. We considered that PEG liposomes enhanced dispersibility by forming a hydrated layer. Optimal particle size facilitates passive targeting using the EPR effect. Regarding the surface charge, our cationic liposomes had a surface charge of ~+50 mV because of cationic lipid DOTAP. The anionic liposomes had a surface charge of ~−40 mV because of CHEMS. In addition, as liposomes are considered a carrier for intravenous administration of drugs, the physical properties of liposomes in the serum are important. Moreover, it is necessary to confirm the absence of liposome aggregation in the serum. However, in this study, the physical properties of liposomes were not evaluated, necessitating future research in this direction. Moreover, the polydispersity index (PDI) was approximately 0.2–0.3 for all liposomes. A PDI value of 0.05 or less is considered ideal for mono-dispersion. Accordingly, the PDI of the liposomes in this study was high. Additionally, as a concern, we assumed that the size or surface charge of each liposome was different. Thus, there is a need to develop methods using a microfluidics method to prepare liposomes of uniform particle size and surface charge for future studies. Moreover, we are planning to confirm more detailed particle physical properties by TEM observation and FT-IR measurement.

The formulations listed in Table 1 can be used to prepare both cationic and anionic liposomes. All four liposomes were intravenously administered to a RA model mouse and qualitatively evaluated by using an in vivo imaging system. Figure 1 shows that the PEG-modified liposome was highly effective at accumulating at the inflammatory sites, and anionic liposome strongly accumulated at the inflammatory sites because of its surface charge. Here, DSPE-PEG2000 was blended at 7.7 or 9 mol% concentration based on the total liposome lipid. In RA model mice, it has been reported that inflammation site accumulation increased with PEG molecular weight while the PEG concentrations (5 mol%, 10 mol%, or 20 mol%) had no influence on this behavior. However, excessively high PEG content may impede drug release and liposome interactions with target cells and ligands [47]. Therefore, we thought that ~10 mol% was designated the optimal PEG content in this study. Based on the aforementioned studies and our results, we proposed that our selected liposome molecular weight and PEG blending quantity were optimal pharmacokinetically speaking [49,50]. Subsequently, anionic liposome accumulation at the inflammation sites was greater than that of cationic liposomes. The renal glomerular basement membrane is negatively charged. Thus, cationic material is far more likely to be excreted than anionic material [51,52]. Moreover, cationic liposomes are readily adsorbed to complement proteins, trapped by RES, and accumulated in the liver. In contrast, anionic liposomes have relatively weaker interactions with blood cell components and non-target organs and more effectively accumulate at the inflammation sites.

Next, we confirmed the efficacy of PEG-modified liposomes in Figure 2. We compared inflammation site accumulation of intravenously administered cationic and anionic PEG liposomes in both CIA and normal mice. Liposome accumulation was never established in healthy mice. In CIA mice, however, liposomes accumulated in their inflamed paws. Furthermore, PEG-anionic liposomes are more strongly accumulated than PEG-cationic liposomes at the inflammation sites. In general, the residence time of PEG-liposomes was approximately 24 h, and although PEG liposomes disappeared after 6 h, which is a relatively short duration. However, we confirmed the superiority of PEG-anionic liposomes for accumulation on inflammatory sites.

PEG modification prevents RES in drugs intravenously administered with nanocarriers. PEG modification reduces the RES trap rate in the liver and spleen and enhances blood retention. Many nano-DDS researched using the EPR effect has been reported for cancer disease. Our results have revealed that a phenomenon resembling the EPR effect occurs at the inflammatory sites of non-tumor tissues. At the inflammatory site, vascular permeability is enhanced by chemical mediators such as histamine and leukotriene. New blood vessels are constructed to repair acute inflammation [53]. In terms of vascular permeability and neovascularization, it is considered that inflammatory tissue is equivalent to cancerous tissue and presents with an EPR effect.

Regarding the difference due to the surface charge of nanocarriers, Ren et al. reported that for a RA mouse model, more intravenously administered anionic than cationic liposomes accumulated at the inflammation sites. In addition, slightly charged liposomes more strongly accumulated at the inflammation sites than heavily charged liposomes. It is reported that slightly anionic liposomes most effectively target arthritis and are best taken up by immunocompetent cells [47,54]. The surface potential of our PEG-modified anionic liposome had a slight charge, and similar results were obtained. Phagocytic cells such as macrophages can phagocytose liposomes with a strong surface charge. In this way, the immune system at the inflammatory site is activated and eliminates them. Thus, the present study indicated that the anionic liposome was more efficacious than the cationic liposome for the intended application for inflammatory site accumulation via intravenous administration.

Evaluation of intravenous nanoparticle accumulation in the ulcerative colitis model mice showed similar results to those observed in the RA mice (Figure 3). It has been reported that when nanoparticles were intravenously administered to DSS-induced mice, particles with an average size of 113 nm accumulated in the large intestine to a greater extent than particles 54 nm or 183 nm in size [55]. Small particles are rapidly released from the inflammation site, while large particles are excluded by RES and fail to accumulate at the inflammation site. The liposomes used in the present study were approximately 50–100 nm, and we considered that our liposomes could easily accumulate. However, PEG-anionic liposomes, which showed the highest accumulation, were approximately 50 nm in diameter, and this differed from the findings of Watanabe et al. [55], necessitating further research. Moreover, it has been reported that intravenously administered nanoparticles with anionic or neutral surface charges more effectively accumulate in inflamed bowels and have comparatively greater therapeutic efficacy [55,56]. However, few studies have compared the relative effectiveness of nanocarriers with different surface charges.

Additionally, referring to lipid selection in this study, the cationic liposomes lacked the cholesterol derivatives present in the anionic liposomes. Thus, anionic liposomes may be more stable than cationic liposomes and accumulate more effectively at the inflammation site. In addition, liposomes with phosphatidylethanolamine groups such as DOPE bind complement C3b, undergo opsonization and are recognized by macrophages and rapidly eliminated from the blood [57,58]. Equal quantities of the cationic and anionic liposomes should be tested and compared. In this study, we established that nanocarriers require PEG modification and anionic charge to reach and accumulate in the target inflammation sites effectively. Both of these factors enhanced inflammatory site accumulation in DSS-induced and CIA mice. In this study, it is indicated that PEG-modified anionic liposomes accumulated in the inflamed tissues to a greater extent than PEG-modified cationic liposomes in the RA and ulcerative colitis model mice. We also assessed the intracellular uptake efficiency of PEG-modified liposomes in immunocompetent cells. Inflammatory disease implicates numerous immunocytes such as dendritic cells, macrophages, and T- and B lymphocytes. Macrophages, in particular, are important in inflammatory disease progression. They are highly active at inflammation sites, produce copious proinflammatory cytokines such as IL-6, IL-1β, and TNF-α, and trigger inflammatory responses [59,60]. Thus, the nanoparticles must deliver the drugs to macrophages at the inflammation sites, and these cells are important targets.

We tested our nanoparticles on RAW264.7 murine macrophages. PEG-cationic liposomes were tenfold more effectively absorbed than PEG-anionic liposomes by RAW264.7 (Figure 4). Previous research and the present study showed that cationic nanocarriers are more effectively uptake than anionic nanocarriers into cells by high electrostatic interactions between positively charged liposomes and the negatively charged cell membranes and cell surface proteoglycans [32,51]. In this study, we have not conducted the experiment on the uptake of non-PEG liposomes, but based on the studies up to Figure 3, PEG modification is indispensable as a carrier for intravenous administration. Therefore, we judged that it was sufficient to confirm only the effect of the charge on the PEG liposome.

Intravenously administered PEG-anionic liposomes were the most efficacious in terms of in vivo accumulation in the inflammatory sites of tumors. Nevertheless, their intracellular uptake capacity was extremely low. Therefore, we propose that PEG-cationic liposomes may effectively target inflammation sites. They align with intracellular target kinetics, accumulate at inflammation sites after intravenous administration, and are readily absorbed by target macrophages. In fact, we have previously confirmed that PEG-modified cationic polymeric micelles accumulated at the inflammation site, suppressed inflammatory cytokine production, and had high therapeutic efficacy after intravenous administration. Therefore, our findings of this study were reasonable.

Finally, in the present study, we evaluated the surface properties of nanoparticles with the accumulation of inflammatory sites by passive targeting using the EPR effect. It is very important to optimize the nanoparticles for passive accumulation. However, we believe that size-controlled passive targeting is incomplete so that nanoparticles must be combined with DDS for enhanced targeting and response to physical stimuli. This modification merits further investigation. Moreover, the absolute values of surface charge may vary for cationic and anionic liposomes. Therefore, we are planning to conduct a dose–effect study on cationic, anionic, and PEGylated liposomes. We will also evaluate the liposomes of uniform charge, particle size, and PDI. The present study focused on the properties required for intravenously administered nanocarriers with optimal drug accumulation and cellular uptake at RA and ulcerative colitis inflammation sites. We confirmed that a phenomenon resembling the EPR effect with intravenous nanoparticles also occurs in inflammatory disease. Based on the in vivo kinetics of inflammation site accumulation, nanoparticle PEG modification and surface charge, and intracellular kinetics, it was confirmed that cationic PEGylated nanoparticles most effectively reach macrophage targets.

## 5. Conclusions

To the best of our knowledge, this is the first study to evaluate the effects of the surface characteristics of nanocarriers on two inflammatory disease sites focused on PEG modification and surface charge. In RA and ulcerative colitis mouse models, intravenously administered PEG-modified anionic liposomes most effectively accumulated in the inflammatory sites. The order was PEG-anionic > PEG-cationic > anionic > cationic. On the other hand, the PEG-cationic liposome was superior in its ability to be taken up by macrophages. Therefore, we propose that PEG-cationic liposomes may effectively target inflammation sites and macrophages by systemic delivery. In future research, we will assess the therapeutic efficacy of PEG-modified cationic nanocarriers encapsulating various drugs. These findings may contribute to enhancing the outcome of their therapy for every inflammatory disease.

## Figures and Tables

**Table 1:** Lipid composition of various liposomes by mol%.

Liposomes	ATTO-DOPE + DOPE (mol%)	DOTAP (mol%)	CHEMS (mol%)	DSPE-PEG_2000_ (mol%)	Total Lipid (µmol)
PEG-cationic	46.15	46.15	-	7.70	6.50
PEG-anionic	54.54	-	36.36	9.10	5.50
Cationic	50.00	50.00	-	-	6.00
Anionic	60.00	-	40.00	-	5.00

**Table 2:** Physical properties of various liposomes.

Liposomes	Particle Size (nm)	Zeta Potential (mV)	PDI
PEG-Cationic	67.8 ± 7.9	+18.9 ± 0.9	0.201 ± 0.008
PEG-Anionic	50.6 ± 0.9	−19.8 ± 1.3	0.309 ± 0.051
Cationic	62.4 ± 2.1	+51.2 ± 0.8	0.231 ± 0.007
Anionic-	86.8 ± 2.4	−42.7 ± 3.5	0.276 ± 0.015

## Data Availability

Not applicable.

## Abbreviations

CFA	Complete Freund’s adjuvant
CHEMS	Cholesteryl hemisuccinate
CIA	Collagen-induced arthritis
CII	Bovine type II collagen
DEPE-PEG2000	2-distearoyl-sn-glycero-3-phosphoethanolamine-N-[methoxy (polyethylene glycol)-2000]
DMEM	Dulbecco’s modified Eagle’s medium
DOPE	1,2-Dioleoyl-sn-glycero-3-phosphoethanolamine
DOTAP	1,2-Dioleoyl-3-trimethylammonium-propane
DSS	Dextran sulfate sodium
EPR	Enhanced permeation and retention
FBS	Fetal bovine serum
IBD	Inflammatory bowel disease
IFA	Incomplete Freund’s adjuvant
PBS	Phosphate-buffered saline
PEG	Polyethylene glycol
RA	Rheumatoid arthritis
RAW264.7	Murine macrophage
RES	Reticuloendothelial system
